# Consumption of soft drinks rich in phosphoric acid versus struvite crystallization from artificial urine

**DOI:** 10.1038/s41598-022-18357-8

**Published:** 2022-08-22

**Authors:** Mikołaj Skubisz, Agnieszka Torzewska, Ewa Mielniczek-Brzóska, Jolanta Prywer

**Affiliations:** 1 Institute of Physics, Lodz University of Technology, ul. Wólczańska 217/221, 93‑005 Łódź, Poland; 2 Department of Biology of Bacteria, Faculty of Biology and Environmental Protection, University of Lodz, ul. Banacha 12/16, 90-237 Łódź, Poland; 3 Institute of Chemistry, Faculty of Science and Technology, Jan Długosz University of Czestochowa, ul. Armii Krajowej 13/15, 42-200 Częstochowa, Poland

**Keywords:** Biophysical chemistry, Biological physics, Bacterial infection

## Abstract

In recent years, there has been a continuous increase in the incidence of urolithiasis, especially in highly developed countries. Therefore, the question arises which factors specific to these countries may be responsible for the increase in the incidence of this disease. In this article, we try to assess the effect of phosphoric acid, a component of various carbonated drinks, including Coca-Cola, on the nucleation and growth of struvite crystals, which are the main component of infectious urinary stones. The research was carried out in the environment of artificial urine with and without the presence of *Proteus mirabilis* bacteria. In the latter case, the activity of bacterial urease was simulated by adding an aqueous ammonia solution. The obtained results indicate that phosphoric acid present in artificial urine causes the nucleation of struvite to shift towards a lower pH, which means that struvite nucleates earlier in artificial urine compared to the control test. The amount of struvite formed is the greater the higher the concentration of phosphoric acid. At the same time, as the concentration of phosphoric acid increases, the growing struvite crystals are larger, which is disadvantageous because they are more difficult to remove from the urinary tract along with the urine. For the highest levels of phosphoric acid tested, large dendrites are formed, which are particularly undesirable as they can damage the epithelium of the urinary tract. The effect of phosphoric acid on the nucleation and growth of struvite is explained in base of chemical speciation analysis. This analysis indicates that the MgHCit and MgCit^−^ complexes have the main influence on the nucleation and growth of struvite in artificial urine in the presence of phosphoric acid. It should be keep in mind that all these effects of phosphoric acid are possible when the urinary tract is infected with urease-positive bacteria. In the absence of infection, phosphoric acid will not cause struvite to crystallize.

## Introduction

**Figure 1:**
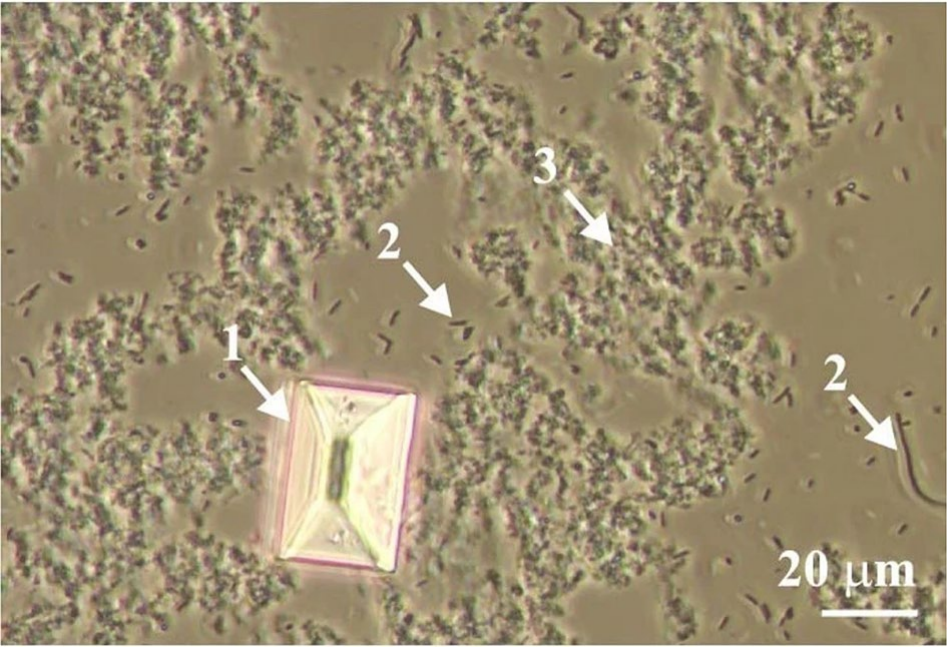
Struvite crystal (1), urease-positive bacteria (2) and other solid phases (PCaAP) (3) in urine^3^.

Infectious urinary stones are easily formed in the urine of animals and humans whose urinary tract is infected with urease-producing microorganisms, which in turn leads to the presence of ammonium ions in the urine. Struvite crystals (magnesium ammonium phosphate hexahydrate; MgNH_4_PO_4_ · 6H_2_O) are the main component of these stones (for example Refs.^1–3^). In the urinary tract of animals and humans infected with urease-positive bacteria, the formation of these crystals is enhanced by alkaline urine and high magnesium excretion (magnesium-rich diets)^4–9^. Chemical reactions in urine in the presence of urease-positive bacteria are described in the literature^10–13^. Struvite crystals usually take the characteristic coffin-shaped habit; Fig. 1, arrow 1. In addition to struvite, other more or less crystalline and/or amorphous solid phases also occur in infectious stones. These phases include, among others: carbonate apatite (CA), hydroxylapatite (HAP), amorphous calcium carbonate (ACC), amorphous calcium phosphate (ACP) and/or amorphous carbonated calcium phosphate (ACCP)^3,14–17^. Generally, these phases are called poorly crystalline and amorphous precipitate (PCaAP)^18^. These phases are marked with arrow 3 in Fig. 1.

In the case of this kind of stones, it is not enough to eliminate the infection itself. Microorganisms can survive in already formed stone by building into its structure. When stones are crushed, for example with high-energy ultrasound waves, microorganisms are released into the urinary tract, which causes disease recurrence after treatment up to 50% of cases^19^. The results to date indicate that even dead microorganisms can be centres of heterogeneous nucleation, accelerating the recrystallization. Additionally, in recent years, an intense increase in the incidence of infectious urinary stones has been observed, especially in populations living in highly developed countries. In these countries, urolithiasis affects up to 20% of the population, depending on the geographical region studied^20–24^.

The question is: what factors are associated with the progressive increase in the incidence of infectious urolithiasis in highly developed countries? In highly developed countries human may be exposed to various adverse factors originating in the environment in which human lives (water, air), originating from food, medications taken and household chemical products. Could soft carbonated drinks intake be such a risk factor for infectious urolithiasis? Consuming carbonated drinks in excess, especially those containing phosphoric acid, such as Coca-Cola, is associated with diseases such as hypertension and diabetes. Daily consumption of this type of beverages also has a real impact on the increased risk of developing chronic kidney diseases^25,26^.

In this article we present studies of the effects of phosphoric acid (H_3_PO_4_), the main ingredient of Coca-Cola and other carbonated drinks, on the nucleation and growth of struvite—the main component of infectious urinary stones. The purpose of these studies is to assess whether the consumption of this type of drink is a risk factor in relation to this type of urolithiasis.

## Materials and methods

### Study of struvite crystallization in artificial urine without bacteria and in their presence

All experiments were performed in an artificial urine environment. The composition of the artificial urine is presented in Table 1.

**Table 1:** Artificial urine composition^27^.

Substance	(g/l)
${\text{CaC}}{\text{l}}_{2}\cdot {2}{\text{H}}_{2}\mathrm{O}$ (calcium chloride dihydrate)	0.651
${\text{MgC}}{\text{l}}_{2}\cdot {6}{\text{H}}_{2}\mathrm{O}$ (magnesium chloride hexahydrate)	0.651
${\text{NaCl}}$ (sodium chloride)	4.6
Na_2_SO_4_ (sodium sulphate)	2.3
Na_3_C_6_H_5_O_7_ (trisodium citrate)	0.65
Na_2_C_2_O_4_ (disodium oxalate)	0.023
KH_2_PO_4_ (potassium dihydrogen phosphate)	2.8
${\text{KCl}}$ (potassium chloride)	1.6
NH_4_Cl (ammonium chloride)	1.0
CH_4_N_2_O (urea)	25.0
C_4_H_7_N_3_O (creatinine)	1.1
Tryptic soy broth (TSB)	10.0

The content of mineral components in such artificial urine corresponds to the average 24-h concentration in the urine of a healthy person and is widely accepted in the literature^3,28^.

As we focused on the nucleation and growth of struvite, we did not add the first ingredient from Table 1, ${\text{CaC}}{\text{l}}_{2}\cdot {2}{\text{H}}_{2}\mathrm{O}$, to the artificial urine. Adding this ingredient would produce PCaAP, the second important component of infectious urinary stones. However, PCaAP would interfere with the ability to objectively assess the progress of struvite crystallization. A detailed description of the formation of struvite and PCaAP in infected urine is presented, for example, in Refs.^3,25,26,29^. Artificial urine was prepared by dissolving reagent grade chemicals (Sigma Aldrich) in deionized water. The solution was then filtered through a membrane filter with a pore size of 0.2 µm. Artificial urine was stored for a maximum of 48 h at 4 °C. The initial pH of the artificial urine was adjusted to 5.8.

The experiment was carried out in two ways: without the presence of bacteria and in the presence of bacteria. In the first case, the presence of bacteria was simulated by adding an aqueous ammonia solution (1.2 M). Such an addition of an aqueous ammonia solution increases the pH and supplies ammonium ions, which closely mimics the decomposition process of urea in the urine by urease-positive bacteria. This procedure was also used previously—for example Refs.^30–32^. The second type of experiment was carried out in the presence of urease-positive bacteria, *Proteus mirabilis* (*P. mirabilis*). The last component of urine from Table 1, i.e., TSB is used to stimulate the bacterial growth. *P. mirabilis* strain was isolated from human kidney stone. Before the experiment, the bacteria were cultured on a tryptic soy agar slant overnight at 37 °C. A suspension of *P. mirabilis* at a concentration of 5 × 10^5^ CFU/ml (the abbreviation CFU/ml stands for colony forming unit per ml) was prepared in the artificial urine and incubated at 37 °C.

The experiments, both in the presence of bacteria and without them, were performed up to a pH in the range of 9–9.5. This is the highest pH that can be achieved with bacteria. In the presence of bacteria, this pH value is obtained after 24 h of the experiment and this value does not increase further^33,34^. The pH of the artificial urine was monitored during the experiments with a digital pH meter (Elmetron CPC-401). With this device, pH measurements are made with an accuracy of 0.01. The paper gives the average results with an accuracy of 0.1. The experiments were carried out in thermostatic conditions at the temperature of 37 ± 0.5 °C. The temperature was kept constant by circulating the water in a constant temperature water bath. Experiments were performed at least in triplicate in order to assess the reproducibility.

The amount of crystallized struvite for the tests with bacteria was evaluated by determining the amount of magnesium. For these analyses, a sample (1 ml) of the crystals with the bacterial suspension was centrifuged at 8000×*g* for 10 min. The obtained pellet was suspended in aqueous solution of nitric acid (30% HNO_3_) and incubated for 60 min at 100 °C. After mineralization, magnesium concentrations were determined by atomic absorption spectroscopy (AAS, SpectrAA-300 Varian, Palo Alto, USA).

### Justification for the selection of the tested concentrations of phosphoric acid

In the presented research, several concentrations of phosphoric acid were examined; Table 2. However, due to the fact that the literature provides reference values for the concentration of phosphorus in urine, and not phosphoric acid, all the tested phosphoric acid concentrations were converted to phosphorus concentrations. Phosphorus, even without the addition of phosphoric acid, is present in the artificial urine because the urine component is KH_2_PO_4_. The phosphorus derived from this component gives the concentration of 20.67 mM. This concentration 20.67 mM is considered as baseline in the present study. The elevated phosphorus concentration tested was equal to 30.45 mM, 43.05 mM, 59.84 mM and 82.66 mM (Table 2, column 3). The phosphorus concentrations presented are the sum of the phosphorus concentrations resulting from the addition of phosphoric acid and the presence of KH_2_PO_4_ in urine. For example, concentration of 30.45 mM means that 9.78 mM comes from phosphoric acid and 20.67 mM comes from KH_2_PO_4_ (Table 2).

**Table 2:** Tested H_3_PO_4_ concentrations equivalent to the corresponding P concentrations.

	H_3_PO_4_ concentration (g/l)	Concentration of P derived from H_3_PO_4_	Total concentration of P derived from H_3_PO_4_ and KH_2_PO_4_
	Column 1	Column 2	Column 3
Baseline, symbol 0	0	0	640 mg/l = 20.67 mM
Concentration 1	0.96	303 mg/l = 9.78 mM	943 mg/l = 30.45 mM
Concentration 2	2.19	693 mg/l = 22.38 mM	1333 mg/l = 43.05 mM
Concentration 3	3.84	1213 mg/l = 39.17 mM	1853 mg/l = 59.84 mM
Concentration 4	6.07	1920 mg/l = 61.99 mM	2560 mg/l = 82.66 mM

### Determination of the viability of bacteria and spectrophotometric measurements

In the case of an experiment in the presence of bacteria, before the crystal growth experiment, viability of bacteria in the presence of phosphoric acid were established. *P. mirabilis* suspension (5 × 10^5^ CFU/ml) was added to wells of microtiter plates containing TSB medium with phosphoric acid in the tested concentrations. Plate was incubated and after 24 h at 37 °C bacterial growth was observed. Acid concentrations at which no growth was observed have a bactericidal effect.

In order to evaluate the effect of increased phosphorus concentration on nucleation and struvite growth, the turbidity of artificial urine with basal and increased phosphorus concentration was measured as the absorbance of light with a specific wavelength. The optimized wavelength was estimated to be 600 nm (for experiments without bacteria and in the presence of bacteria). The absorbance was measured with a Schimadzu 2600 (in the absence of bacteria) and Ultrospec 2000 (Pharmacia Biotech) spectrophotometer (in the presence of bacteria) using cuvettes with an optical path length of 10 mm. During all experiments, samples were taken at regular intervals and observed under an Opta Tech MN 800 (in the absence of bacteria) and Nikon Eclipse TE2000-S (in the presence of bacteria) optical microscope to assess the progression of struvite crystal growth, habit, and size.

### Theoretical analysis of the influence of phosphoric acid on the formation of chemical complexes in artificial urine

In order to explain the influence of phosphoric acid on the nucleation and growth of struvite crystals in artificial urine, a theoretical analysis of the chemical complexes formed in urine was performed, both in the presence of this acid and without it. This analysis was performed using the HySS (Hyperquad Simulation and Speciation) software^35^. Using the equilibrium constants of complexes formation (stability constants), and equilibrium constants of sparingly soluble salts (solubility product constants) and by introducing the initial molar concentrations of ions constituting the complexes in artificial urine, the software allows the calculation of molar concentrations of various species in a given pH range. With this software, we can analyse the effects of phosphoric acid on the formation of chemical equilibriums, and on the formation of struvite in an artificial urine. The stability constants used in the HySS program were calculated using the EQUIL computer code^36^. The calculations were performed for the temperature of 37 °C.

## Results

### Effect of phosphoric acid on nucleation and growth of struvite in the absence of urease-positive bacteria

#### Spectrophotometric analysis

In the presented research, several concentrations of phosphoric acid were examined; Table 2. However, due to the fact that the literature provides reference values for the concentration of phosphorus in urine, and not phosphoric acid, all the tested phosphoric acid concentrations were converted to phosphorus concentrations. Phosphorus, even without the addition of phosphoric acid, is present in the urine because the urine component is KH_2_PO_4_. The phosphorus derived from this component gives the concentration of 20.67 mM. This concentration 20.67 mM is considered as baseline in the present study (symbol 0). The elevated phosphorus concentration tested was equal to 30.45 mM (symbol 1), 43.05 mM (symbol 2), 59.84 mM (symbol 3) and 82.66 mM (symbol 4). The phosphorus concentrations presented are the sum of the phosphorus concentrations resulting from the addition of phosphoric acid and the presence of KH_2_PO_4_ in urine.

The starting point for the selection of such phosphorus concentrations was the recommended, average, permissible and maximum phosphorus intake per day. The tested concentrations 1, 2, 3 and 4 (Table 2) result from the recommended, average, permissible and maximum phosphorus consumption, respectively. The recommended consumption of phosphorus in healthy people over 18 years of age, regardless of sex, including breastfeeding and pregnant women, is 700 mg^37^ per day, regardless of body weight. According to the 2015–2016 research conducted by the National Health and Nutrition Examination Survey (NHANES)^38^, the average daily intake of phosphorus by adult women is 1190 mg, and for men 1600 mg. We also took into account the so-called permissible daily phosphorus intake (the maximum amount of a substance per kilogram of body weight that can be consumed daily throughout life without significant negative health effects), which is 40 mg per kilogram of body weight. The maximum daily consumption of phosphorus is 70 mg per kilogram of body weight^39^. In the presented research, we assumed that we are considering a human weighing 70 kg, which, when calculating the permissible and maximum consumption, gives 2800 mg and 4900 mg of phosphorus per day, respectively. When determining phosphorus concentrations for research, it was necessary to determine the method of converting the ingested phosphorus into the amount that ultimately ends up in the urine after it is used by the human digestive system. According to Ref.^40^, in a person with properly functioning kidneys, the amount of phosphorus ingested is equivalent to the phosphorus excreted in urine and faeces. According to the standards of digestion in a healthy person, 35% of ingested phosphorus is excreted in the faeces, and 65% in the urine^41^. In the calculations, we assumed that a healthy person excretes 1.5 l of urine daily. The concentrations of phosphorus 1, 2, 3 and 4 presented in Table 2 are converted according to the above standards and are given per litre of urine.

**Figure 2:**
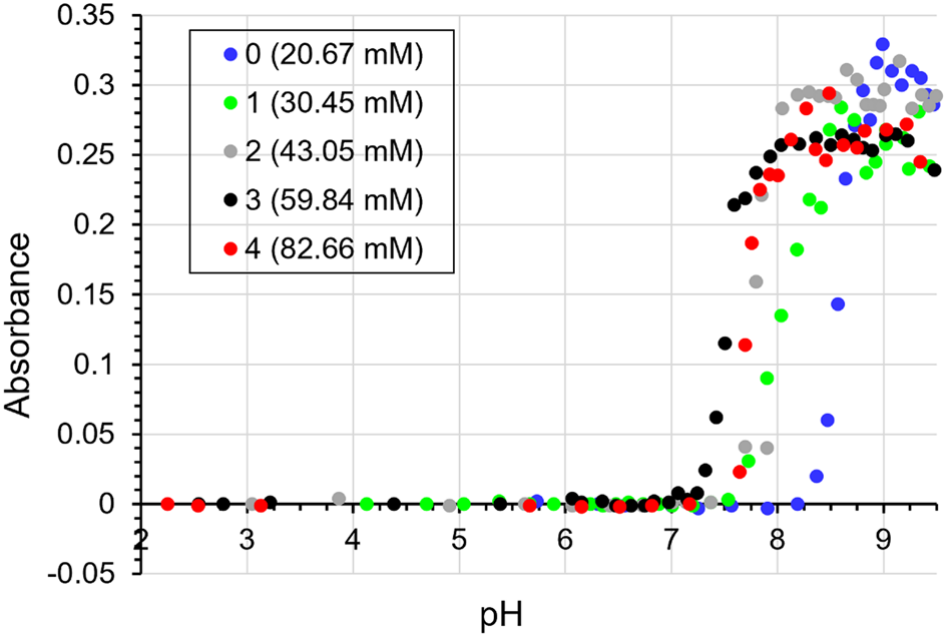
Dependence of absorbance (measured at a wavelength of 600 nm) on the pH of artificial urine for the baseline concentration of phosphorus 20.67 mM (symbol 0) and for the increased concentration of phosphorus 30.45 mM, 43.05 mM, 59.84 mM, and 82.66 mM—symbols 1, 2, 3 and 4, respectively.

In order to characterize the nucleation and growth processes of struvite, changes in the turbidity of artificial urine were measured in the absence of phosphoric acid (baseline) and in the presence of phosphoric acid with concentrations presented in Table 2. First, we present the results obtained without the presence of bacteria. In this case, in order to mimic the activity of bacteria, and more specifically the activity of bacterial urease, a 1.2 M aqueous ammonia solution was added in small portions (6 μl) to the artificial urine. Consequently, as described in paragraph “Materials and methods” as well as repeatedly in the literature^10–13^, the pH of the artificial urine increases, and the process of struvite nucleation begins, which is manifested by the progressive turbidity of this solution. The measure of this turbidity is the absorbance. The dependence of artificial urine absorbance on pH for baseline and tested phosphorus concentrations is shown in Fig. 2.

There are some interesting points to note in Fig. 2. First, it can be seen that phosphoric acid lowers the initial pH of the urine. The higher the phosphoric acid concentration, the lower the initial pH. Specifically, the initial pH values are 5.68, 4.20, 3.15, 2.51 and 2.20 for the phosphorus concentrations 20.67 mM (baseline), 30.45 mM, 43.05 mM, 59.84 mM, and 82.66 mM, respectively. The mechanism of the lowering of the urine pH under the influence of phosphoric acid is explained in paragraph  “Analysis of the results from the point of view of the formation of chemical complexes in artificial urine"

The second thing to note is that Fig. 2 shows that phosphoric acid shifts the appearance of struvite towards lower pH. This is evidenced by the fact that in the presence of phosphoric acid, a sudden increase in absorbance corresponding to the appearance of struvite occurs at lower pH values compared to the control test (baseline). In the case of baseline, the increase in absorbance, indicating the appearance of struvite crystals in the urine, occurs at a pH of 8.2 (Fig. 2). In the case of phosphorus concentration equal to 1, the increase in absorbance occurs for pH = 7.6. In the case of phosphorus concentrations 2, 3 and 4, the increase in absorbance occurs at even lower pH values (Fig. 2). However, there is no linear relationship between phosphoric acid concentration and the lowering pH at which struvite appears. This means that phosphoric acid with a concentration 4 (the highest) does not significantly lower this pH compared to, for example, a concentration 3.

**Figure 3:**
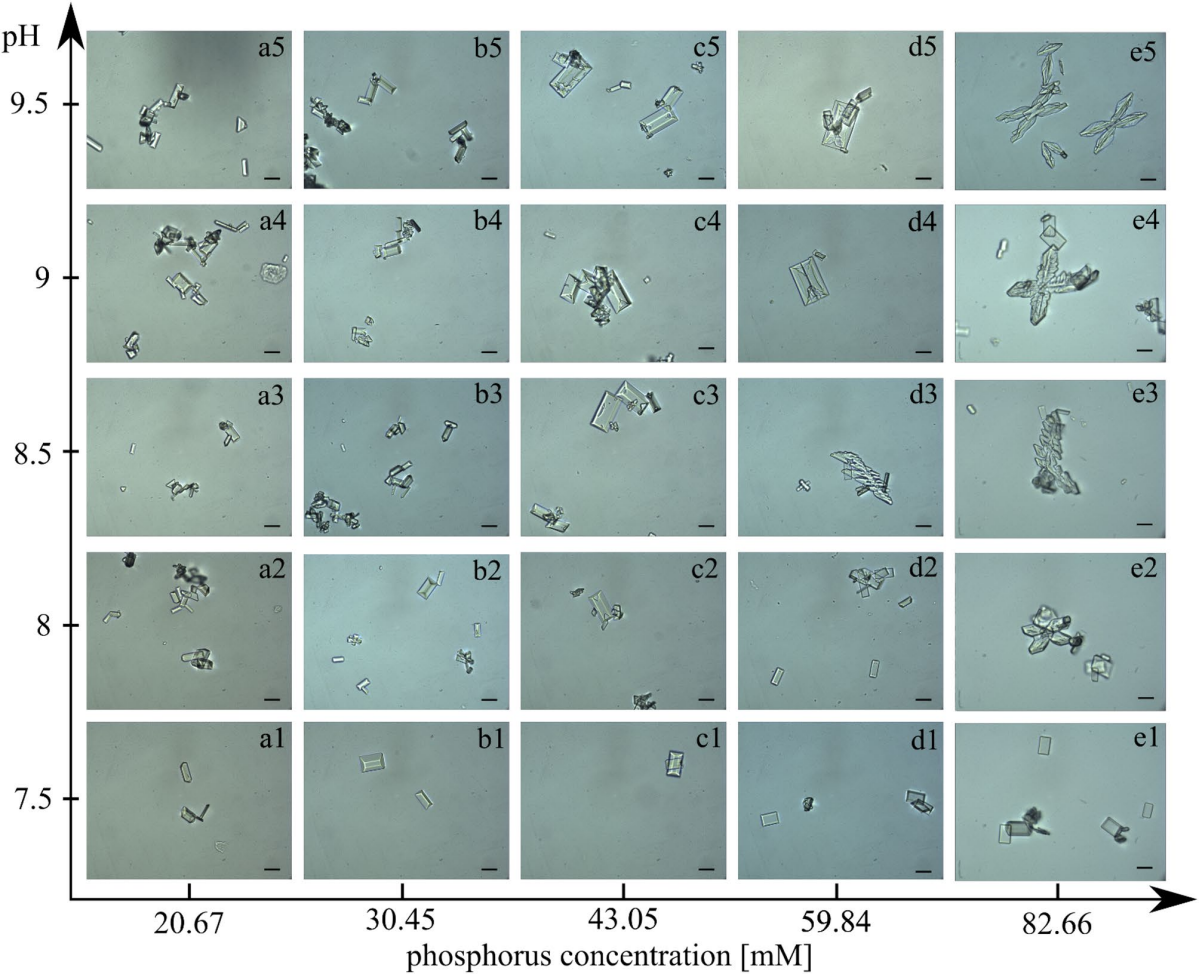
The course of struvite crystals growth in artificial urine with different phosphorus concentration and increasing pH. Scale bar 10 µm.

**Figure 4:**
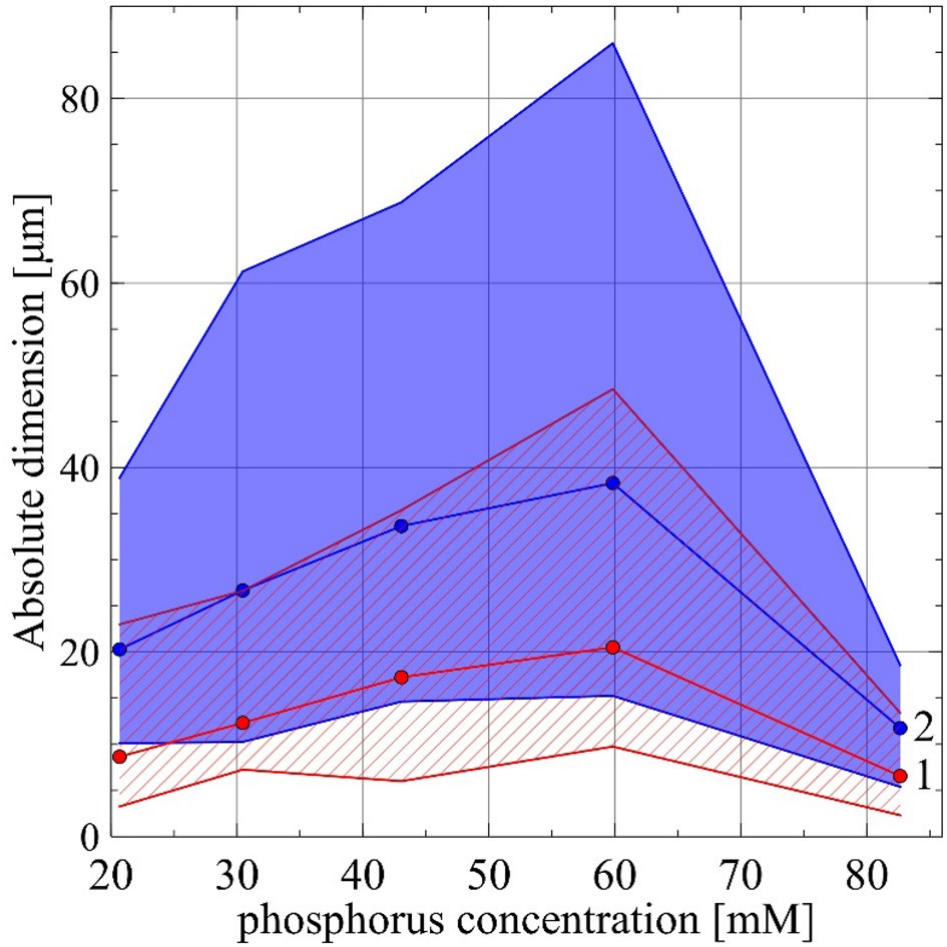
Dependence of the absolute dimensions of struvite crystals along the *a*-axis (1) and along the *b*-axis (2) on the concentration of phosphorous in artificial urine. The solid lines represent the mean values of the dimensions measured, while the shaded and lined areas correspond to span between the measured smallest and largest dimensions.

These spectrophotometric absorbance measurements are confirmed by microscopic observations (Fig. 3). In the case of baseline, we see the first single crystals for pH 7.5 (Fig. 3, panel a1). For this pH, there are so few of these crystals that they give no increase in absorbance. For higher pH, the amount of struvite crystals increases (Fig. 3, panels a2–a5), while their size does not increase significantly (the analysis of the crystal size is presented later in this paper). Importantly, no dendrites appear either (Fig. 3, panel a2–a5). The habit of the crystals is coffin-shaped, which is consistent with the description in the literature^42–47^. For phosphorus 1 concentration, the course of struvite growth is similar to that for baseline (Fig. 3, panels b1–b5). Except that the crystals are larger (Figs. 3, 4) but preserve their coffin-shaped habit (Fig. 3, panels b1–b5). For phosphorus concentration 2, the struvite crystals are significantly larger (Fig. 3, panels c1–c5 and Fig. 4), but still preserve the characteristic coffin-shaped habit. The course of the growth process for the concentration of phosphorus equal to 3 (Fig. 3, panels d1–d5) is slightly different compared to the lower concentrations and baseline. In this case, in addition to the large coffin-shaped crystals (Fig. 3, panels d1–d5), we observe the first large dendrites (Fig. 3, panel d3). For this concentration, individual struvite crystals show the largest size (Fig. 4). The situation is different for the highest phosphorus concentration tested, equal to 82.66 mM. In this case, practical from the beginning, we observe large dendrites, mainly X-shaped (Fig. 3, panels e1–e5). The presence of dendrites may indicate high growth dynamics and a high growth rate. These dendrites are accompanied by single coffin-shaped struvite crystals, but of a much smaller size compared to the lower phosphorus concentrations tested. For a phosphorus concentration of 82.66 mM, the size of the non-dendritic struvite crystals is comparable to the crystal size of the baseline (Fig. 4).

**Figure 5:**
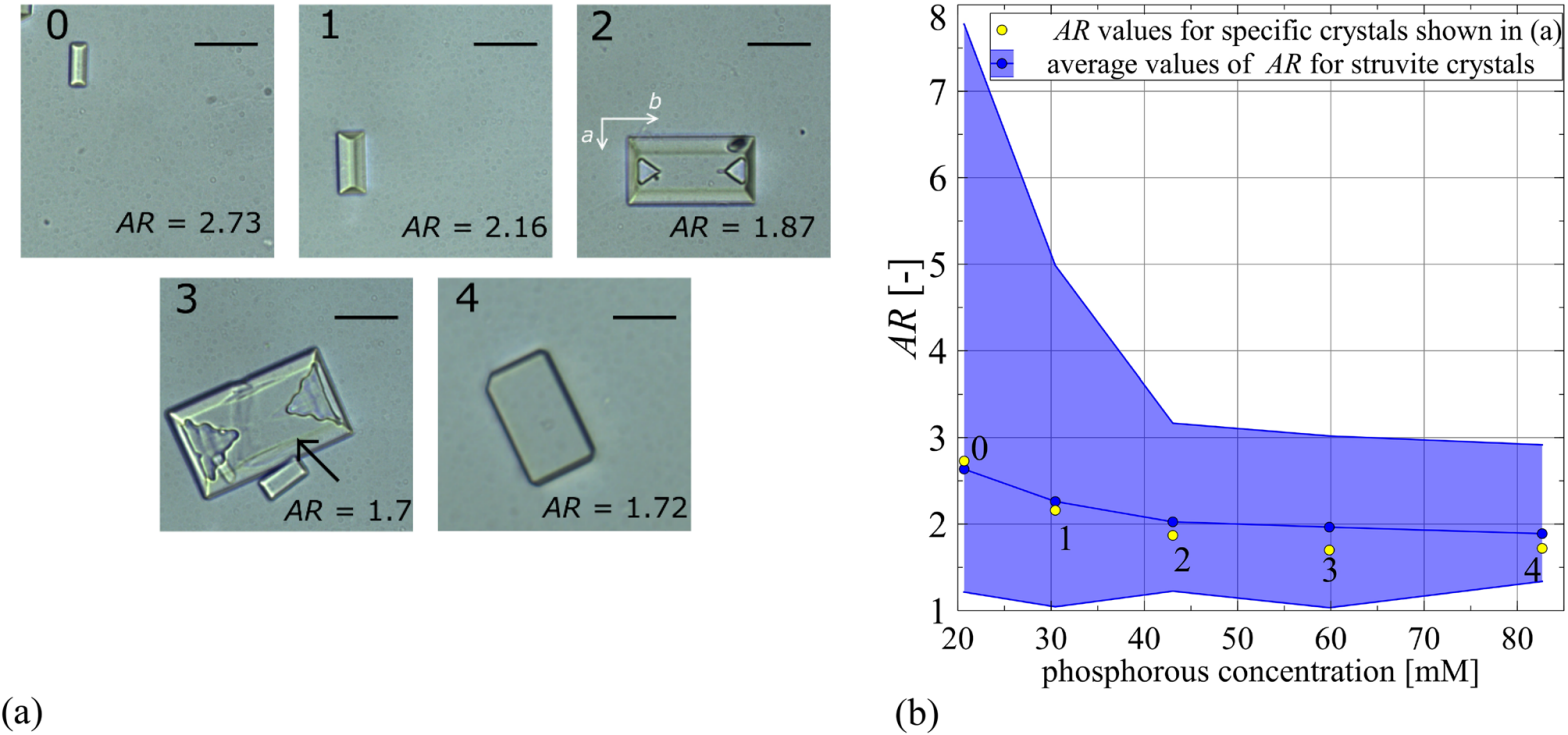
(**a**) Change in *AR* of struvite crystals depending on phosphorus concentration in artificial urine for baseline (20.67 mM)—symbol 0 and for increased phosphorus concentration: 30.45 mM, 43.05 mM, 59.84 mM, and 82.66 mM—symbols 1, 2, 3 and 4, respectively. In case 3, *AR* is calculated for the crystal indicated by the arrow. Scale bar 10 µm. (**b**) Dependence of the aspect ratio (*AR*) of struvite crystals on the concentration of phosphorus in artificial urine. The solid line represents the mean values of the dimensions measured, while the shaded area represents the span from the smallest to the largest measured values. The numbers refer to the numbering of the photos in (**a**) and correspond to the concentrations of phosphorous.

In addition to the absolute dimensions, with increasing phosphorus concentration, the aspect ratio, *AR*, defined as the crystal length ${l_{b} }$ along the *b*-axis to the length ${l_{a} }$ along the *a*-axis, also changes. The lengths ${l_{b} }$ and ${l_{a} }$ are defined in Fig. 5a, panel 2 (for all the crystals shown in Fig. 5a the longer side of the crystal is along the *b*-axis and the shorter side along the *a*-axis). From Fig. 5a,b, it can be seen that *AR* is the highest for baseline and it gradually decreases for increasing phosphorus concentration. Even the lowest tested phosphorus concentration causes a significant decrease in *AR*, and for the highest tested concentrations, 59.84 mM, and 82.66 mM (Fig. 5; symbols 3 and 4), *AR* reaches the lowest value, which for these both concentrations is comparable. Such a significant change in *AR* taking place under the influence of phosphorus acid means that this acid significantly influences the relative growth rates of individual crystal faces. The crystals shown in Fig. 5a, panels 2 and 3 show inclusions of the mother solution. Their existence can be explained by the relatively high relative growth rates of individual faces.

The third interesting thing that can be seen from the dependence of absorbance on increasing pH (Fig. 2) is that the maximum absorbance (for pH around 9) decreases with increasing phosphoric acid concentration. For example, the absorbance value at pH 9 for baseline is about 0.31 and for the lowest phosphorus concentration tested of 30.45 mM it is 0.25. For higher phosphorus concentrations, the maximum absorbance values for pH 9 are equal to 0.27, 0.24 and 0.23 for concentrations of 43.05 mM, 59.84 mM, and 82.66 mM, respectively. This could indicate that the amount of struvite formed decreases with increasing phosphoric acid concentration. However, this conclusion is not supported by the weighing of the samples. After the end of the experiment, all samples were centrifuged and, after pouring out the artificial urine, the resulting crystals were weighed. Table 3 shows the struvite masses for an exemplary, one measurement series.

**Table 3:** Final (for pH 9.5) mass of struvite (mg) for all tested concentrations of P, for an exemplary one measurement series.

	struvite mass (mg) for pH 9.5	The percentage (%) increase in weight relative to the baseline
Baseline 0	16.3	100
Concentration 1	22.2	136
Concentration 2	18.2	112
Concentration 3	22.1	136
Concentration 4	22.1	136

As shown in Table 3, the presence of phosphoric acid in the sample increases the amount of struvite formed. The mass of the formed struvite is greater compared to baseline but practically comparable for all phosphoric acid concentrations. Therefore, the observed decrease in the maximum absorbance above pH 8.5 (Fig. 2) in the presence of phosphoric acid is rather due to the fact that for the samples with phosphoric acid the amount of aqueous ammonia added is much greater. This is because in the presence of phosphoric acid, the initial pH is lower compared to the baseline. Therefore, in the case of samples with phosphoric acid, more aqueous ammonia must be added to raise the pH to 9.5. The absorbance decreases for samples with phosphoric acid because the struvite formed is dispersed in a larger sample volume (the volume increases as an increased amount of aqueous ammonia is added). The amount of aqueous ammonia solution added to all samples for an exemplary one experimental series is given in Table 4. The initial volume of the samples is 25 ml.

**Table 4:** The amount (ml) of added aqueous ammonia solution for samples with an initial volume of 25 ml for one selected measuring series.

	Added aqueous ammonia solution (ml)
Baseline 0	1.43
Concentration 1	2.00
Concentration 2	2.96
Concentration 3	4.48
Concentration 4	5.91

In conclusion, the results of the studies indicate that phosphoric acid in the urine (in the absence of bacteria) causes a decrease in the initial pH of the urine. In the presence of this acid, in urine, in which the presence of urease-positive bacteria is simulated by adding aqueous ammonia solution, nucleation and growth of struvite occurs earlier (for a lower pH) compared to the control sample (without the presence of phosphoric acid). The amount of struvite formed in the presence of phosphoric acid is greater compared to the control test. However, this amount does not increase in proportion to the increasing concentration of this acid. For the highest phosphorus concentration tested, struvite occurs in the form of large dendrites that can damage the epithelium of the urinary tract. Phosphoric acid also affects the relative growth rates of the individual faces of struvite crystals, as evidenced by the change in the *AR* coefficient for the increasing concentration of this acid.

#### Analysis of the results from the point of view of the formation of chemical complexes in artificial urine

Numerous dissociation, hydrolysis and complexing reactions take place in artificial urine solution with both normal and elevated P concentrations. As a result, different chemical equilibria between formed species are achieved. The analysis of the speciation of the complexes in artificial urine in the presence of phosphoric acid was performed using the HySS computer code^35^. The purpose of the analysis is to determine the dominant chemical form of the complexes for baseline (0) and in the presence of phosphoric acid at the tested concentrations (1, 2, 3 and 4). Knowledge of the structure and stability of complexes in growth solution containing phosphoric acid is essential for understanding the kinetics of struvite crystal growth. In the speciation analysis, we focus in particular on Mg^2+^, PO_4_^3−^ and NH_4_^+^ ions, as they are the constituent ions of struvite.

Table 5 presents the initial concentrations of individual ions, resulting from the composition of artificial urine and, from the tested concentrations of P. In the absence of bacteria, their presence is simulated by adding an aqueous solution of ammonia as described in paragraph  “Spectrophotometric analysis”. Table 4 gives the amount of added 1.2 M aqueous ammonia solution. The amount of aqueous ammonia solution added may influence the concentration of the ions presented in Table 5 due to the fact that the initial volume of the samples (25 ml) slightly increases. Therefore, the amount of ammonia solution added was taken into account when calculating the concentrations of individual ions presented in Table 5. Table 6 presents the concentrations of all these ions for all tested concentrations of P after adding the amount of aqueous ammonia solution, i.e., their final concentrations. On the basis of Table 6 it is possible to notice a slight decrease in the molar concentration of individual ions, resulting from the addition of an aqueous ammonia solution, i.e., from a slight dilution of the samples.

**Table 5:** Initial concentrations, *c*, of particular ions taken into account in the HySS calculations.

species	Ca^2+^	Mg^2+^	C_2_O_4_^2−^	Cit^3−^	SO_4_^2−^	Na^+^	K^+^	NH_4_^+^	P
*c* (mM)	0^a^	3.2	0.2	2.5	16.2	126.3	42.0	800^b^	20.67^c^
									30.45^c^
									43.05^c^
									59.84^c^
									82.66^c^

^a^In this study we used artificial urine with modified composition without calcium chloride dihydrate which results in an initial Ca^2+^ concentration of 0 mM.

^b^This amount of NH_4_^+^ results from the assumption that the entire available amount of urea (25 g/l, Table 1) is decomposed to form, inter alia, NH_3_ in gaseous form (details in Ref.^48^).

^c^These are the phosphorus concentrations tested in this study—see Table 2, column 3. Cit stands for [C_6_H_5_O_7_] and results from the presence of trisodium citrate in the artificial urine.

**Table 6:** Final concentrations, *c*_end_, after adding aqueous ammonia solution (Table 4), of the individual ions included in the HySS calculations, for all tested P concentrations (0, 1, 2, 3 and 4).

Species	*c*_end_ (mM)				
	Presence of bacteria simulated by adding aqueous ammonia solution				
	0^a^	1^a^	2^a^	3^a^	4^a^
P	19.55	28.19	38.49	50.74	66.85
C_2_O_4_^2−^	0.19	0.18	0.18	0.17	0.16
Cit^3−^	2.38	2.28	2.21	2.11	2.00
SO_4_^2−^	15.32	14.80	14.31	13.70	12.93
Mg^2+^	3.03	2.92	2.83	2.71	2.55
Na^+^	119.47	115.41	111.55	106.80	100.80
K^+^	39.73	38.38	37.10	35.51	33.52
NH_4_^+^	756.72	740	720	678.43	647.04

^a^These P concentrations (0—baseline and 1–4 tested concentrations) are investigated in this article. The calculations took into account the addition of an aqueous ammonia solution (Table 4), which resulted in a slight change in the molar concentrations of individual ions compared to the initial ones (Table 5).

**Figure 6:**
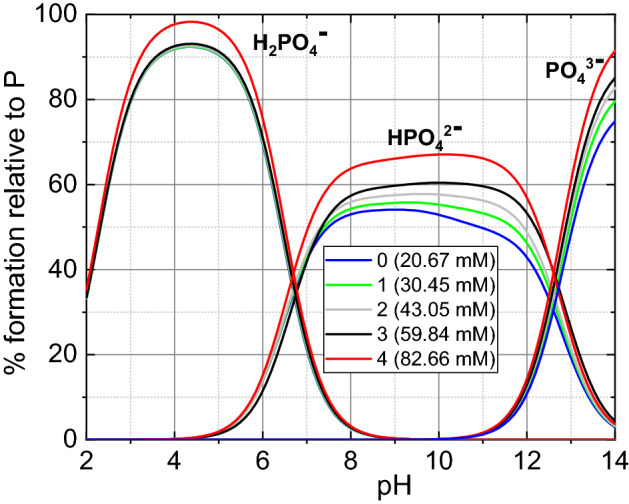
Percentage content of PO_4_^3−^ ions and their complexes (HPO_4_^2−^ and H_2_PO_4_^−^) depending on pH of the artificial urine for different concentrations of P given in the inset.

**Figure 7:**
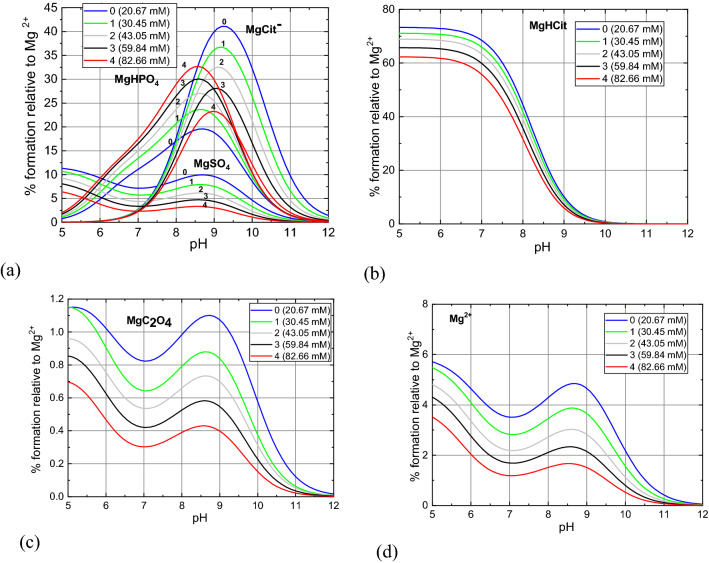
Percentage content of various Mg^2+^ complexes (**a**–**c**) and free Mg^2+^ ions (**d**) depending on pH of artificial urine for different P concentrations. The percentage content is given with respect to the initial concentration of Mg^2+^ ion given in Table 5.

**Figure 8:**
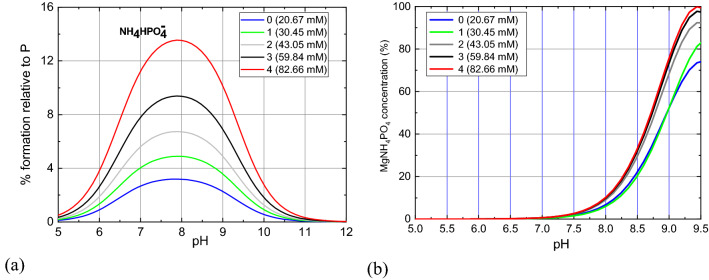
Percentage content of different complexes (**a**) NH_4_HPO_4_^−^ and (**b**) MgNH_4_PO_4_, formed with NH_4_^+^ ions versus pH of artificial urine for different P concentrations given in the inset.

After entering the initial and final concentrations of individual ions (Table 5, 6, respectively) and the stability constants and solubility product constants of the formed complexes (Table 7) into the HySS computer program, the appropriate molar and percentage concentrations of the chemical complexes in the given pH range were calculated. The results of the analysis, i.e., the concentration of the dominant complexes in the artificial urine for baseline and with the addition of phosphoric acid are shown in Figs. 6, 7 and 8.

**Table 7:** Stability constants –log*β* of chemical complexes which are taken into account in the analysis.

Complex	− log*β*	Complex	− log*β*
HPO_4_^2−^	12.6	MgC_2_O_4_	3.62
H_2_PO_4_^−^	19.3	Mg_2_C_2_O_4_^2+^	4.28
H_3_PO_4_	21.56	Mg(C_2_O_4_)_2_^2−^	4.38
NaHPO_4_^−^	13.2	NH_4_C_2_O_4_^−^	1.11
KHPO_4_^−^	13.3	H_2_C_2_O_4_	5.68
MgCit^−^	4.67	MgOH	3.16
MgHCit	13.07	Mg_4_OH_4_^4+^	16.43
MgH_2_Cit^+^	12.43	KOH	1.5
MgHPO_4_	15.16	HSO_4_^−^	2.14
MgPO_4_^−^	5.88	NaSO_4_^−^	0.61
MgH_2_PO_4_^+^	20.53	KSO_4_^−^	0.997
NH_4_HPO_4_^−^	13.16	NH_4_SO_4_^−^	0.67
MgNH_4_PO_4_	0.0043	NH_4_OH	6.732
KMgPO_4_	1	MgSO_4_	2.407
Mg_3_(PO_4_)_2_	1	KC_2_O_4_^−^	1.2
HCit^2−^	6.46	KCit^2−^	1.24
H_2_Cit^−^	11.16	NH_4_Cit^2−^	0.085
H_3_Cit	14.49	HC_2_O_4_^−^	4.32
NaCit^2−^	1.35	NaC_2_O_4_^−^	0.995

The presented stability constants are calculated using computer code EQUIL^36^ at 37 °C.

The first thing is that speciation analysis can explain the mechanism of phosphoric acid lowering urine pH. When an appropriate amount of H_3_PO_4_ acid is added to the urine, dissociation occurs, and the following chemical equilibria are formed between the chemical individuals (Fig. 6):

1 $${\text{H}}_{3} {\text{PO}}_{4} \leftrightarrow {\text{H}}_{2} {\text{PO}}_{4}^{ - } + {\text{H}}^{ + } \quad K_{{{\text{a1}}}} = { 5}.{5} \times {1}0^{{ - {3}}}$$

2 $${\text{H}}_{2} {\text{PO}}_{4}^{ - } \leftrightarrow {\text{HPO}}_{4}^{2 - } + {\text{H}}^{ + } \quad K_{{{\text{a2}}}} = { 2}.0 \times {1}0^{{ - {7}}}$$

3 $${\text{HPO}}_{4}^{2 - } \leftrightarrow {\text{PO}}_{4}^{3 - } + {\text{H}}^{ + } \quad K_{{{\text{a3}}}} = { 2}.{5} \times {1}0^{{ - {13}}}$$

where *K*_a1_, *K*_a2_ and *K*_a3_ are the dissociation constants calculated using computer code EQUIL^36^ for the temperature of 37 °C, due to the fact that the experiment was carried out at this temperature. In the literature, these constants are usually given for 25 °C (for example Ref.^49^), but their values are comparable to those for 37 °C. It can be seen from the above equations that such individuals as ${\mathrm{H}}_{2}{\mathrm{PO}}_{4}^{-}$, ${\mathrm{HPO}}_{4}^{2-}$, ${\mathrm{PO}}_{4}^{3-}$ and hydrogen ions are formed. In Fig. 6 we can see that with an increase in the concentration of phosphoric acid, the concentration of individuals such as ${\mathrm{H}}_{2}{\mathrm{PO}}_{4}^{-}$, ${\mathrm{HPO}}_{4}^{2-}$, ${\mathrm{PO}}_{4}^{3-}$ also increases. This means that the concentration of hydrogen ions also increases, as evidenced by urine pH measurements. The pH value decreases when phosphoric acid is added, and the higher the concentration, the lower the pH. In the case of baseline (Fig. 6, symbol 0), the initial urine pH is around 5, which means that forms such as ${\mathrm{H}}_{2}{\mathrm{PO}}_{4}^{-}$, ${\mathrm{HPO}}_{4}^{2-}$ dominate, and after adding phosphoric acid, mainly reactions (1) and (2) take place.

As indicated by experimental studies, with an increase in phosphoric acid concentration, struvite begins to crystallize at slightly lower pH values and, additionally, the amount of struvite formed slightly increases. In order to explain these facts, one should analyse the Figs. 7 and 8 showing the percentage content of different complexes formed with Mg^2+^ and NH_4_^+^ ions. Analysis of the modified urine (i.e. without calcium ions) using the HySS computer program shows that with an increase in the concentration of phosphoric acid in urine from 0.96 g/l to 6.07 g/l  (Table 2, column 1), the following changes compared to urine without the addition of phosphoric acid (1), the concentration of such complexes as MgHCit (Fig. 7b), ${\mathrm{MgCit}}^{-}$(Fig. 7a), MgSO_4_ (Fig. 7a) and MgC_2_O_4_ (Fig. 7c) decreases, (2) the concentration of complexes such as MgHPO_4_ (Fig. 7a), ${\mathrm{NH}}_{4}{\mathrm{HPO}}_{4}^{-}$ (Fig. 7d) increases significantly. These observations suggest that in the pH range from 5 to 9, the MgHCit (Fig. 7b), ${\mathrm{MgCit}}^{-}$(Fig. 7a), MgSO_4_ (Fig. 7a) and MgC_2_O_4_ (Fig. 7c) complexes are mainly responsible for a large increase in the concentration of MgHPO_4_ complexes. In other words, a large number of MgHPO_4_ complexes are formed at the expense of MgHCit, ${\mathrm{MgCit}}^{-}$, MgSO_4_ and MgC_2_O_4_ complexes. Figure 8 shows that in the presence of phosphoric acid, slightly more struvite is formed, and the formation of struvite shifts slightly towards lower pH, which is consistent with experimental observations. It seems that the increase in the amount of struvite formed in the presence of phosphoric acid, based on the presented speciation analysis, can be explained by the fact that the presence of this acid increases the amount of complexes such as ${\mathrm{HPO}}_{4}^{2-}$, and decreases the amount of complexes such as Mg^2+^, ${\mathrm{MgCit}}^{-}$ and MgHCit. As the amount of these complexes is decreasing in the pH range characteristic of struvite growth, this means that these complexes are involved in struvite formation. Therefore, in the presence of phosphoric acid, the following reactions of struvite formation can be proposed:

4 $${\mathrm{Mg}}^{2+ }+{\mathrm{HPO}}_{4}^{2-}+ {\mathrm{NH}}_{4}^{+}\to {\mathrm{MgNH}}_{4}{\mathrm{PO}}_{4 }+{\mathrm{H}}^{+}$$

5 $${\mathrm{MgCit}}^{- }+{\mathrm{HPO}}_{4}^{2-}+ {\mathrm{NH}}_{4}^{+}\to {\mathrm{MgNH}}_{4}{\mathrm{PO}}_{4 }+{\mathrm{HCit}}^{2-}$$

6 $${\mathrm{MgHCit}}^{- }+{\mathrm{HPO}}_{4}^{2-}+ {\mathrm{NH}}_{4}^{+}\to {\mathrm{MgNH}}_{4}{\mathrm{PO}}_{4 }+{\mathrm{H}}_{2}{\mathrm{Cit}}^{-}$$

The amount of ${\mathrm{HPO}}_{4}^{2-}$ complexes increases and the amount of Mg^2+^, ${\mathrm{MgCit}}^{-}$ and MgHCit complexes decreases successively with increasing phosphoric acid concentration. This means that as the concentration of phosphoric acid increases, more and more struvite is formed according to reactions (4)–(6). This tendency is illustrated in Fig. 8b and is consistent with the experimental observations (Table 3).

### Effect of phosphoric acid on nucleation and growth of struvite in the presence of urease-positive bacteria

#### Spectrophotometric analysis

**Figure 9:**
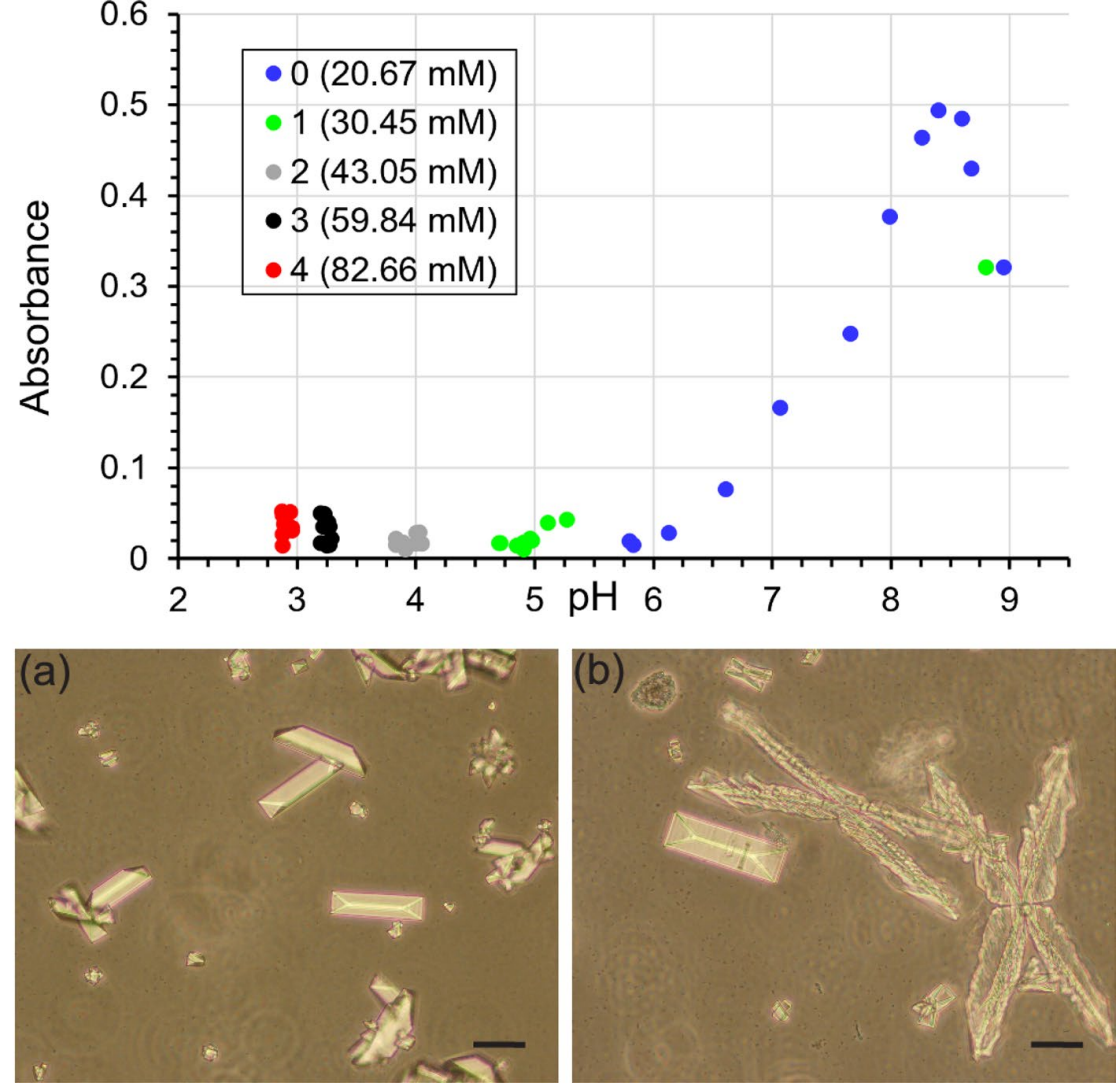
Absorbance (measured at a wavelength of 600 nm) of artificial urine versus pH for various P concentrations (top row) in the presence of bacteria. Bottom row: (**a**) habit of struvite crystals in the control system (baseline), and (**b**) for concentration 1 (30.45 mM), after 24 h incubation. Scale bar 40 µm.

In the presence of bacteria, the progress of struvite nucleation and growth was monitored for the first 8 h after incubating the bacteria in artificial urine and then 24 h after this incubation. The results of spectrophotometric measurements are presented in Fig. 9.

**Table 8:** The amount of NaOH added to the samples with phosphoric acid concentration 2, 3 and 4 in order to raise their initial pH to the initial pH level for the sample with phosphoric acid concentration 1.

H_3_PO_4_ concentration	The amount of 2 M NaOH added (µl)	Initial pH–adjusted pH
2	180	3.85–4.78
3	360	3.25–4.71
4	620	2.79–4.74

As can be seen from Fig. 9 (top row), the absorbance increases successively in the case of baseline (without phosphoric acid; symbol 0). For concentration 1, we see an increase in absorbance after 24 h (absorbance for pH 8.8). In both of these cases, struvite crystallization took place (Fig. 9a,b). However, for concentrations of 2, 3 and 4, the absorbance practically does not increase, even after 24 h. After determining the killing effect of phosphoric acid at the tested concentrations, it turned out that only concentration 1 was not killing bacteria. For the remaining phosphoric acid concentrations tested, the initial pH of the artificial urine is too low for bacterial growth. Due to the lack of bacterial growth at higher concentrations of phosphoric acid, there is no change in pH and absorbance value.

**Figure 10:**
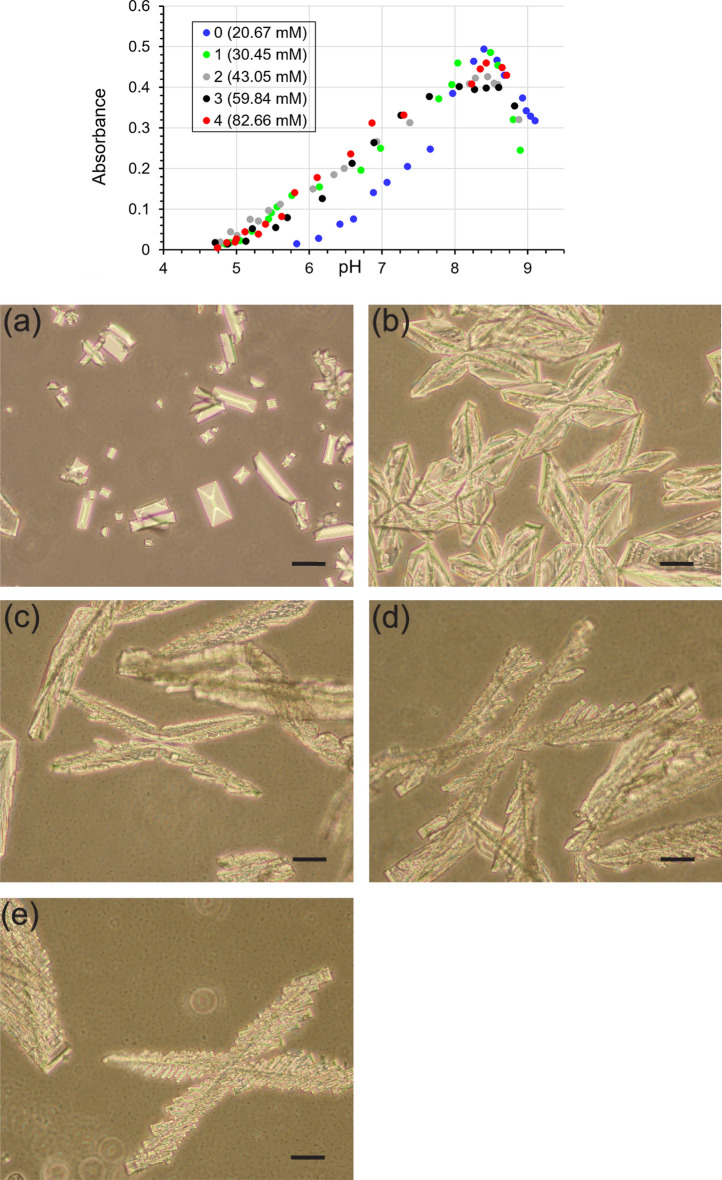
Absorbance (measured at a wavelength of 600 nm) of artificial urine versus pH for various P concentrations (top row) in the presence of bacteria (after the initial urine pH has been raised to approximately 4.7). Habit of struvite crystals in the control system (baseline)—(**a**), and for concentration 1 (**b**), 2 (**c**), 3 (**d**) and 4 (**e**), 24 h after adding bacteria to urine. Scale bar 40 µm.

Due to the killing effect of such a low pH caused by phosphoric acid, we decided to increase the initial pH in the samples with concentrations of 2, 3 and 4 using 2 M NaOH to the pH value of the sample for concentration 1, i.e., 4.7. The amount of added NaOH is shown in Table 8. This addition of NaOH to raise the pH to the initial pH level for a concentration of 1 of H_3_PO_4_ makes sense because such low pH values ​​of the order of 2, 3 seem unrealistic for the human urinary system. In a healthy person, there is a continuous flow of urine in the urinary tract, the person also ingests various fluids during the day and some of this urine is excreted. This means that the pH can be modified in the living organism. The presented experiment is carried out in vitro, in stationary conditions without urine flow. For the experiment designed in this way, bacterial growth took place for all tested phosphoric acid concentrations, which resulted in nucleation and growth of struvite crystals (Fig. 10).

Figure 10 (top row) shows that the crystallization processes are shifted towards lower pH, similar to the case without bacteria. Figure 10a–e show the struvite crystal habit. It can be seen that, in the control test (Fig. 10a), the struvite takes on a characteristic coffin-shaped habit, similar to that in the absence of bacteria. This habit has been described many times in the literature^42–47^. When phosphoric acid is present (Fig. 10b–e), struvite appears as large dendrites. For higher concentrations of P, struvite also appears as dendrites, but we do not present photos as they would be analogous to those in Fig. 10a–e. Dendrites are also observed in the case of the experiment without the presence of bacteria (Fig. 3).

It is also worth asking whether the addition of NaOH to raise the initial pH of samples with phosphoric acid in concentrations of 2, 3 and 4 does not affect the concentrations of individual chemical complexes (analysis in paragraph “Analysis of the results from the point of view of the formation of chemical complexes in artificial urine”). In order to test this problem, we counted the concentrations of the individual ions after adding the appropriate amounts of NaOH given in Table 8. These concentrations are given in Table 9. As shown in Table 9, the greatest changes in concentration concern Na^+^, which is not surprising as NaOH was added. Changes in the concentrations of the remaining complexes, including those of key importance for struvite formation (Mg^2+^, NH_4_^+^, PO_4_^3−^), are very small (compared to the baseline), therefore we consider these changes negligible. Therefore, the speciation analysis presented in “Analysis of the results from the point of view of the formation of chemical complexes in artificial urine” also applies to experiments carried out in the presence of bacteria.

**Table 9:** The initial concentrations, *c*, of the individual complexes, taking into account the addition of small volumes of 2 M NaOH (see Table 8) to the samples with P concentrations of 2, 3 and 4, compared to the initial state (concentration 0).

Species	*c* (mM)	*c* (mM)	*c* (mM)	*c* (mM)	*c* (mM)
	0	1	2^a^	3^a^	4^a^
P	20.67	30.45	43.0	59.47	82.15
C_2_O_4_^2−^	0.20	0.20	0.20	0.20	0.20
Cit^3−^	2.52	2.52	2.52	2.51	2.46
SO_4_^2−^	16.20	16.20	16.17	16.14	15.81
Mg^2+^	3.20	3.20	3.19	3.19	3.12
Na^+^	126.30	126.30	129.67	133.02	137.85
K^+^	42.0	42.0	41.92	41.85	40.98
NH_4_^+^	800.0	800.0	798.56	797.13	795.10

The concentrations for 0 and 1 are identical because no NaOH was added to samples 0 and 1.

^a^The calculations took into account the added small amounts of 2 M NaOH (Table 4), which resulted in a slight change in the initial molar concentrations of individual ions compared to samples 0 and 1.

The question remains whether the amount of struvite formed increases with the increase in phosphoric acid concentration. In order to estimate the amount of struvite formed, we cannot weigh the precipitate in the case of an experiment with bacteria, as in the case of the absence of bacteria. This is mainly due to the fact that in case of weighing the bacteria would also contribute as there are no easy methods to separate the bacteria from the sediment. Therefore, in the case of bacteria, in the control sample and in samples with different phosphoric acid concentrations, we determined the amount of Mg (Table 10). The amount of Mg indicates the amount of struvite. From Table 10 it can be concluded that there are no differences in the amount of Mg between the control and H_3_PO_4_ samples. It should be noted that the value of the measurement uncertainty (Table 10) is standard, considering that that we are dealing with a biological system^50^. In conclusion, it can be concluded that the amounts of struvite formed for all tested samples are comparable or slightly different. However, it should also be noted that in the absence of bacteria, the amount of formed struvite increases with the increase in phosphoric acid concentration, but it is not a very large increase (Table 3).

**Table 10:** Determined amount of Mg in samples in the presence of bacteria after 24 h of the experiment.

	Mg (µg/ml)				
	H_3_PO_4_ concentration				
	0	1	2	3	4
Average amount	67.02	65.98	67.42	63.8	68.64
measurement uncertainty	4.28	24.58	24.53	23.4	24.24

## Conclusions

In this paper, we present the effect of phosphoric acid on the crystallization of struvite, the main component of infectious urinary stones. We tested four concentrations of phosphoric acid 1, 2, 3 and 4. The obtained results show that phosphoric acid in urine leads to several significant changes compared to the control test. First, phosphoric acid lowers the pH of the urine. For concentrations of this acid 2–4, the decrease in the initial urine pH is so great that no struvite crystallization occurs at all, due to the fact that such a low pH (in the range of 3.85–2.79) is lethal for bacteria. And that is, of course, a positive effect. However, it should be borne in mind that the presented studies were conducted *in vitro*, without a continuous flow of urine. In the urinary tract of a healthy person, the decrease in urine pH may not be as high. After increasing the urine pH with NaOH to a non-bactericidal level, the higher the phosphorus concentration, the earlier (for a lower pH) the struvite crystallization process begins, which is obviously not a favourable effect. The test results also show that the amount of crystallizing struvite slightly increases with the increase in phosphoric acid concentration. In the case of an experiment in the presence of bacteria, this effect is imperceptible. In both cases, in the absence of bacteria and in their presence, large dendrites are formed in the presence of phosphoric acid, especially for higher concentrations of this acid, which is not a favourable effect.

The above experimental observations related to the presence of phosphoric acid in urine were elucidated on the basis of theoretical speciation analysis of chemical complexes formed in urine. This analysis indicates that the MgHCit and MgCit^−^ complexes have the main influence on the nucleation and growth of struvite in artificial urine in the presence of phosphoric acid.

It should be remembered that all these effects of phosphoric acid are possible in the case of urinary tract infection with urease-positive bacteria. In the absence of infection, phosphoric acid will not cause struvite to crystallize.

It is also worth paying attention to one issue, namely what happens when the patient already has a urinary stone made of, for example, calcium oxalate, not related to urinary tract infection with urease-positive bacteria. In this case, in the context of the research presented in this article, three factors would influence the formation and growth of urinary stone: urinary tract infection, increased levels of phosphorus in the urine, and the presence of a urinary stone. In this situation, it is very likely that the existing urinary stone could build up faster. This is due to the fact that the surface of the urinary stone is usually porous, which favours the adhesion of bacteria, which may become nucleation centres for subsequent layers of the urinary stone. Typically, urinary stone has a multi-layered structure with a different chemical composition^51^.
